# Decrease in Ribosomal RNA in *Candida albicans* Induced by Serum Exposure

**DOI:** 10.1371/journal.pone.0124430

**Published:** 2015-05-06

**Authors:** Jacob Fleischmann, Miguel A. Rocha

**Affiliations:** 1 Department of Medicine, Greater Los Angeles VA Healthcare System, Los Angeles, California, United States of America; 2 Research Division, Greater Los Angeles VA Healthcare System, Los Angeles, California, United States of America; 3 Department of Medicine, David Geffen School of Medicine at UCLA, Los Angeles, California, United States of America; Worcester Polytechnic Institute, UNITED STATES

## Abstract

*Candida albicans* is an important polymorphic human pathogen. It can switch from a unicellular yeast form to germinating hypha, which may play a role in making it the successful pathogen it is. This hyphal transformation can be triggered by various extracellular stimuli, the most potent one being serum from any source. We have previously reported that *Candida albicans* transiently polyadenylates portions of both the large and small subunits of ribosomal RNA, shortly after serum exposure. Northern blots at the same time suggested that serum might induce a decrease in total ribosomal RNA. We have carried out a number of experiments to carefully assess this possibility and now report that serum significantly reduces ribosomal RNA in *Candida albicans*. Fluorometric measurements, Northern blotting and quantitative RT-PCR, have all confirmed this decrease. Timed experiments show that serum induces this decrease rapidly, as it was seen in as early as five minutes. Cell mass is not decreased as total cellular protein content remains the same and metabolic activity does not appear to slow, as assessed by XTT assay, and by the observation that cells form hyphal structures robustly. Another hyphal inducer, N-acetylglucosamine, also caused RNA decrease, but to a lesser extent. We also observed it in non-germinating yeast, such as *Candida glabrata*. The reason for this decrease is unknown and overall our data suggests that decrease in rRNA does not play a causal role in hyphal transformation. Rapid and significant decrease in a molecule so central to the yeast’s biology is of some importance, and further studies, such as its effect on protein metabolism, will be required to better understand its purpose.

## Introduction

*Candida* species are important pathogens especially for immunocompromised patients. They are the fungal species most commonly recovered from normally sterile sites in these patients [1]. Among the *Candida* species that cause human disease, variable phenotypic behaviors are observed. These range from polymorphic changes exhibited to external signals by *Candida albicans*, to *Candida glabrata* which remains in a yeast form exclusively [2]. Yet, clinically they cause similar pathology in the patients they infect. The signal transduction pathways underlying yeast to hyphal transformation have been most intensively studied in *Candida albicans* and a number of these are now well established, including some with negative regulatory activity [3, 4]. It was interesting, that as gene components of these pathways were being identified and their deletions indeed showed impairment in yeast to hypha transition by various stimuli, serum exposure was still able to induce hyphal formation in these deleted yeasts [5, 6, 7]. This led us to look for possible additional genes, whose transcriptions were activated shortly after serum exposure. Unexpectedly, we found that portions of both the large and the small ribosomal RNA (rRNA) subunits were polyadenylated transiently just prior to the phenotypic appearance of hypha [8, 9]. At that time we found, but not yet reported on, that while our poly-A selected Northern blots showed an early and transient increase of both the small and large subunits of rRNA shortly after serum exposure, unselected Northern blots simultaneously showed a significant decrease in total rRNA, reaching a nadir as early as 30 minutes post treatment. We have now extended on these observations and report indeed a significant and rapid decrease in rRNA after serum exposure prior to the appearance of hypha.

## Materials and Methods

### Organisms and germination

*Candida albicans* SC5314 (purchased from ATCC MYA 2876) was maintained in 50% glycerol in YPD broth (2% w/V tryptone, 1% w/v yeast extract, 2% w/v dextrose) at -80°C. Cells were activated in YPD broth at 30°C and maintained on Sabouraud dextrose agar at 4°C, passaged every 4–6 weeks up to 4–5 times. Additional yeasts were received from the Greater Los Angeles VA System microbiology laboratory. These included *Candida krusei* ATCC 14243, *Candida tropicalis* ATCC 750, *Candida glabrata* MYA 2950 and a *Candida dubliniensis* WLA 7.15 a clinical isolate identified by Vitek 2 (Biomerieux) and ChromAgar Candida (Hardy Diagnostics). Yeast were lifted from agar surface and grown in YPD broth for 16 hours at 30°C. Yeast cell concentrations were established using a hemocytometer. 5 X 10^7^ cells were collected by centrifugation, washed with sterile phosphate buffered saline (PBS) and were put on ice to await total RNA extraction (referred to as T0 min). For germination assays again 5 X 10^7^ cells were suspended in 50 ml of pre-heated (37°C) 10% v/v heat inactivated fetal bovine serum (FBS) in sterile double distilled water for a final concentration of 1 X 10^6^ cells ml^-1^. Additional assays with other media included water alone, undiluted FBS, fresh YPD broth alone, YPD broth with 10% (v\v) FBS and 2% (w\v) *N*-acetyl-D-glucosamine (GlcNAc). All of these assays had yeast concentration of 1 X 10^6^ cells ml^-1^ and the same 50 ml volumes at 37°C. The length of assays was as reported in the result sections. It has also been reported for *C*. *albicans*, that change in temperature alone from 37°C to 39°C in YPD induces hyphal transformation [10]. We also set up assays following the reported parameters. Cells were grown for 16 hours at 37°C in YPD. Enough cells were transferred to 25 ml YPD heated to 39°C for a final concentration of 2 X 10^7^ ml^-1^. At the same time a second group of cells same in number were put on ice (T0). After 1 hour total RNA was extracted from both groups and measured as above. Germ tube formations were followed microscopically for all assays.

### RNA extraction and measurement

After incubation for germination, cells were collected with centrifugation at 5°C, washed with cold PBS and had RNA extracted simultaneously with T0 min cells. Total RNA isolation was done by using the components and the protocol of the RiboPure Yeast RNA Purification Kit (Life technologies). This included cell disruption with RNAse-free zirconium beads in the presence of RNAse inhibiting lysis solution and column purification. The isolated RNA was treated with RNAse-free DNAse I. To insure that another mode of isolation gave us similar results as the kit, RNA was extracted simultaneously once, using the hot acid phenol isolation protocol [11]. RNA quantification was done on total freshly isolated RNA, always diluted in the same volume of 50 μL, using a Qubit 2.0 fluorometer. To see if any polyadenylated rRNAs are involved, we did a one time poly-A selection at times T0 T30 T60 utilizing the BcMag Quick mRNA Purification Kit (Bioclone Inc.) according to the manufacturer’s directions.

### Viability assay

To insure that cell viability is not a confounding factor, XTT viability assays were performed simultaneous to RNA extraction [12]. Yeast cells were suspended in 5 ml volume of the same solution with the same conditions as used in those from which RNA was extracted. Fresh assay working solution was prepared each time as follows. A XTT sodium salt (Sigma-Aldrich) solution at 1 mg ml^-1^ was made in pre-heated (37°C) PBS. 370 μl of this solution was combined with 30 μl of 1 mM menadione (Sigma-Aldrich) previously prepared in acetone. Yeast cells were collected by centrifugation, washed with PBS and suspended in the 400 μl of working solution in 96-well plates. Plates were incubated at 37°C with moderate shaking. To measure formazan derivative formation, spectrophotometric readings at 490 nm were obtained at appropriate times, using the Epoch Micro-plate Spectrophotometer (BioTek).

### Protein extraction and measurement

To exclude significant loss in cell mass, protein was extracted using Y-PER Yeast Protein Extraction Reagent (Thermo Scientific). Yeast numbers, germination conditions and timing were the same as those for the RNA extraction and XTT experiments. After the incubation periods, cells were pelleted at 3000 x g for 5 minutes at 4°C. The pellets were suspended in 250 μL Y-PER reagent and vortexed gently at room temperature for 20 minutes. The mixture was centrifuged at 14,000 x g for 10 minutes saving the supernatant containing the soluble protein. The protein concentration was measured with the Qubit 2.0 fluorometer using the Qubit Protein Assay Kit (Life Technologies).

### Northern blotting

RNA was separated on pre-fabricated formaldehyde agarose gels (Lonza) and stained with SYBR Gold Nucleic Acid Gel Stain (Life Technologies) for 30 minutes. Gel images were captured with a digital camera (Canon Vixia HFS30). RNA was transferred by electro-blotting (Thermo Scientific Owl Hep-1) to a positively charged nylon membrane (Life Technologies) in 0.5 x TBE (standard Tris/Borate/EDTA buffer). The RNA was cross-linked to the membrane using UV (Stratagene UV Crosslinker). Probes for the 25S, 18S, and 5S components of the ribosomal RNA gene of *Candida albicans* SC5314 were synthesized (Blue Heron Gene Synthesis Co.) (Table 1) and cloned into Blue Heron pUC. Plasmids were inserted into EC100 E. coli (Epicentre Biotech) and stored at -80°C. For probe preparation, bacteria were grown in Terrific Broth (Fisher) with ampicillin at 50 μg ml^-1^, and plasmids were isolated with a QIAprep Spin Miniprep Kit (Qiagen). Inserts were released with *Bam*H I/*Eco*R I for 25S, 5S, and *Bam*H I/*Nco* I for 18S, and were purified with a QIAquick Gel Extraction Kit (Qiagen). 50–100 ng of purified inserts were biotinylated using the BrightStar Psoralen-Biotin Nonisotopic Labeling Kit (Life Technologies). Membranes were pre-hybridized at 55°C in North2South hybridization buffer (Pierce) for 30 min followed by overnight hybridization with denatured biotinylated probes at 5ng ml^-1^ in the same buffer at 55°C. Membranes were washed with low and high stringency buffers at room temperature and RNA was detected using Chemiluminescent Nucleic Acid Detection Kit (Pierce) according to the manufacturer’s protocol. Film was developed with the SRX-101A Konica film processor. Northern blot and Sybr Gold images were densometrically analyzed by ImageJ software from NIH.

**Table 1:** Probe Sequences for Non-Isotopic Labeling.

18S	Chromosomal Coordinates *
AAATCTTGTGAAACTCCGTCGTGCTGGGGATAGAGCATTGTAATTGTTGCTCTTCAACGAGGAATTCCTAGTAAGCGCAAGTCATCAGCTTGCGTTGATTACGTCCCTGCCCTTTGTACACACCGCCCGTCGCTACTACCGATTGAATGGCTTAGTGAGGCCTCCGGATTGGTTTAGGAA	1898043–1898223
**25S**	
AGGAACCGTTCATTCAGATAATTGGTTTTTGCGGCTGTCTGATCAGGCAACGCCGCGAAGCTACCATCTGCTGGATTATGGCTGAACGCCTCTAAGTCAGAATCCATGCTAGAACGCGATGATTTTTGCCCTGCACATTTTAGATGGATACGAATAAGACTTTTTAGTCGCTGGACCATA	1896543–1896723
**5S**	
GGTTGCGGCCATATCTAGCAGAAAGCACCGTTCCCCGTTCGATCAACCGTAGTTAAGCTGCTAAGAGCAATACCGAGTAGTGTAGTGGGAGACCATACGCGAAACTATTGTGCTGCAATCT	1889007–1889127

* http://www.candidagenome.org/

### Quantitative PCR

RT-PCR. Quantitative Real Time PCR was performed in the CFX Connect Detection System (Bio-Rad) using the QuantiTect SYBR Green (Qiagen) kit. Total RNA was isolated from the same number of yeast cells as described above. The same volume of total RNA for each time point was diluted one hundred fold and one μL of diluted RNA was used for the cDNA synthesis and amplification for each time point and subunit. Primers (Table 2) were designed with the NIH primer design tool and synthesized commercially by Eurofins Genomics (Louisville, KY). The reactions parameters were as follows: 55°C for 30 minutes, 96°C for 3 minutes, followed by 40 one-minute cycles of 96°C, 68°C for 5 minutes, 72°C for 10 minutes and finally cooled to 4°C. The number of amplicons generated in each reaction were analyzed with the CFX Manager Software (Bio-Rad) and presented as the ratio of amplicons generated at T0 to either T30 or T60 for each subunit.

**Table 2:** Primer sequences for RT-PCR.

**18S-Fwd**	Chromosomal Coordinates *
5’-AACGGCTACCACATCCAAGG-3’	1891654–1891824
**18S-Rev**	
5’-CACCAGACTTGCCCTCCAAT-3’	
**25S-Fwd**	
5’-CCAACAGGGATTGCCTCAGT-3’	1893501–1893716
**25S-Rev**	
5’-CCCTCTGTGACGTCCTGTTC-3’	
**5S-Fwd**	
5’-GGTTGCGGCCATATCTAGCA-3’	1889206–1888965
**5S-Rev**	
5’-TTTGATAACACGACGTTAGA-3’	

http://www.candidagenome.org/

### Statistics

Standard deviations and P-values using 1-sample T test were calculated on Excel.

## Results

### Total RNA in 10% serum/H_2_O stimulation

**Fig 1:**
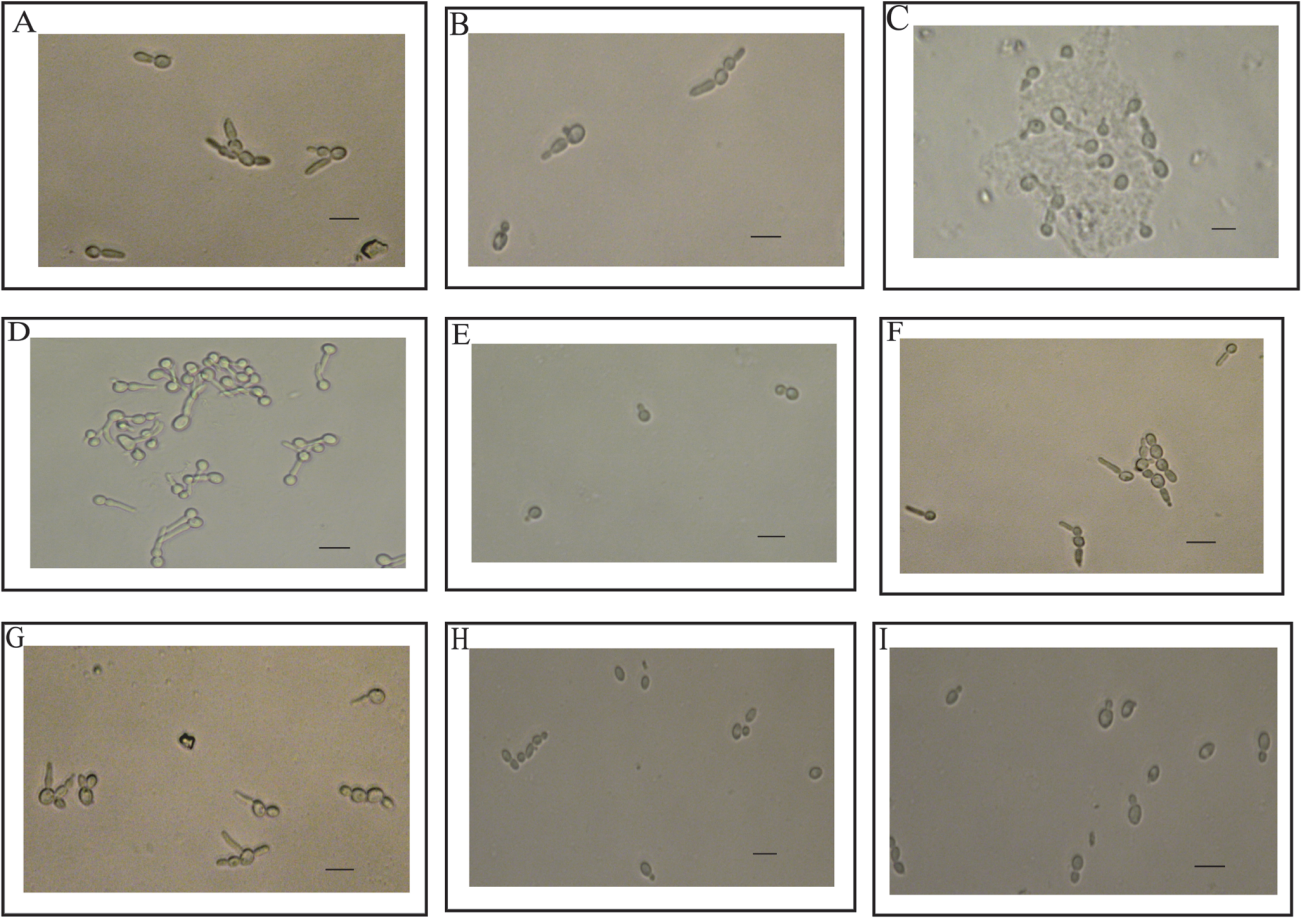
Yeast cells exposed to various solutions. A. *C*. *albicans* in water plus 10% FBS for 30 min. B. *C*. *albicans* in YPD plus 10% FBS for 30 min. C. *C*. *albicans* in 100% FBS for 30 min. D. *C*. *albicans* in water plus 10% FBS for 60 min. E. *C*. *albicans* in water for 60 min. F. *C*. *dubliniensis* in water plus 10% FBS for 60 min. G. *C*. *tropicalis* in water plus 10% FBS for 60 min. H. *C*. *glabrata* in water plus 10% FBS for 60 min. I. *C*. *krusei* in water plus 10% FBS for 60 min. All assays were done at 37°C. Pictures were taken at 40X magnification. The bars represent 10μm.

**Fig 2:**
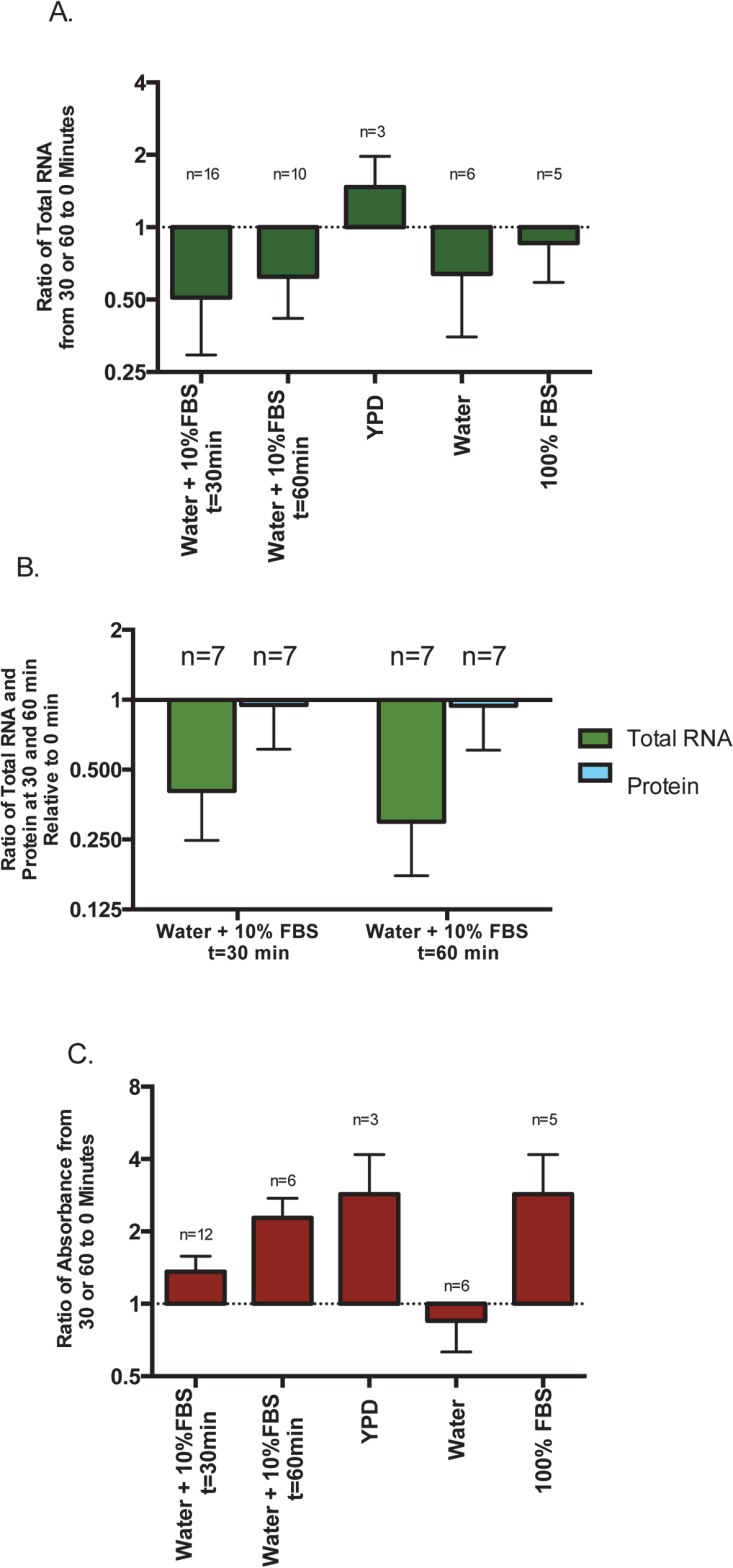
Effect of hyphal stimulation on total cellular RNA in *Candida albicans* and simultaneous assessment of protein content and of viability by XTT assay. A. Bars represent ratios of total RNA after stimulation to total RNA prior to stimulation. Error bars represent SD. Assays lasted 30 minutes except where otherwise noted. Significant decreases of RNA were seen with water plus 10% FBS for 30 min (p = 0.0004), same assay for 60 min (p = 0.01) and water alone (p = 0.001). FBS alone also showed decrease but it did not reach significant levels. YPD alone increased RNA. B. Protein extraction run simultaneously with RNA extraction from cells exposed to water plus 10% FBS for 30 and 60 minutes. Bars represent ratios of total protein or RNA after stimulation to those prior to stimulation. Error bars represent SD. Protein content remains stable while RNA content is decreased. C. XTT assays run simultaneously with conditions mirroring those of part A. Bars represent ratios of absorbance after stimulation to those before stimulation. Error bars represent SD. All except water show increase indicating no loss in viability and increasing metabolic activity.

Ten percent serum in water remains among the most potent stimuli for *Candida albicans* to shift from yeast to hypha. By 30 minutes, after transfer into 10% serum at 37°C, hypha can be easily seen (Fig 1A). As seen on Fig 2A, total RNA is significantly decreased with more than half of the assays showing a decrease of more than fifty percent. Viability is clearly not the underlying reason for this decline, as formazan formation remains intact, in fact increasing at 30 minutes (Fig 2C). Results at 30 minutes with each of the three solutions used individually are also seen in Fig 2A and 2C. Using fresh YPD with only a change in the temperature from 30°C to 37°C, did not result in any hyphae and both RNA content and formazan formation increased (Fig 2A and 2C). Water alone without any other nutritional source at 37°C did not induce hypha (Fig 1E) and showed small declines both in RNA and formazan (Fig 2A and 2C). Using 100% serum at 37°C, significant clumping by the organisms can be seen (Fig 1C) in addition to hyphal formation. Total RNA was decreased but to a lesser extent as compared to 10% serum (Fig 2A), yet formazan formation was as robust as YPD alone (Fig 2C). A number of 10% serum assays were carried out to 60 minutes (Fig 2A and 2C). Extensive hyphae were observed (Fig 1D) and formazan increase (Fig 2C) mirrored this, yet total RNA remained decreased similar in level to 30 minutes (Fig 2A). Loss in cell mass was also not the reason for rRNA decrease as extracted protein remained similar in all three time points (Fig 2B). Poly-A selection of total RNA extracted at T0, T30 and T60, in one experiment yielded amounts below 2% of the total RNA, indicating that RNA isolated was non-polyadenylated. The one time comparison of RNA extraction with hot acid phenol showed the same significant decrease in RNA as the kit did. Data available from the Dryad Digital Repository [13].

### Specific RNAs in 10% serum/H_2_O stimulation

**Fig 3:**
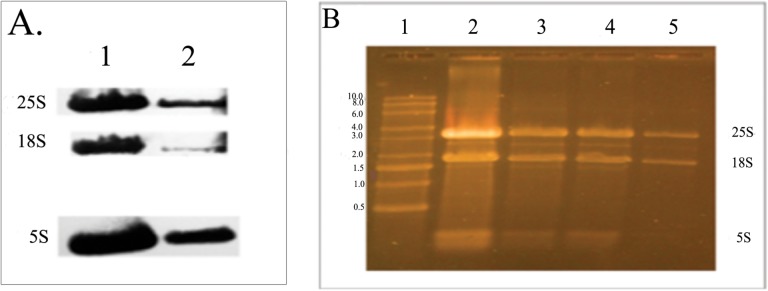
Northern blot analysis for ribosomal RNAs from *Candida albicans* comparing before and after hyphal stimulation by water with 10% FBS. A. Each lane had 5 μL loaded from the 50 μL volume containing the total extracted RNA (see text). Lane 1 was from unstimulated cells (T = 0). Lane 2 from cells exposed to water plus 10% FBS for 30 min (T = 30) at 37°C. Nylon membrane was probed sequentially with probes indicated on Table 1. B. Gel used for blotting seen in part A, stained with SYBR Gold stain. Lane 1 has RNA size markers. RNA loaded for other wells are as follows: Lane 2–5μL T = 0, Lane 3–5μL T = 30, Lane 4–1μL T = 0, Lane 5–1μL T = 30.

**Fig 4:**
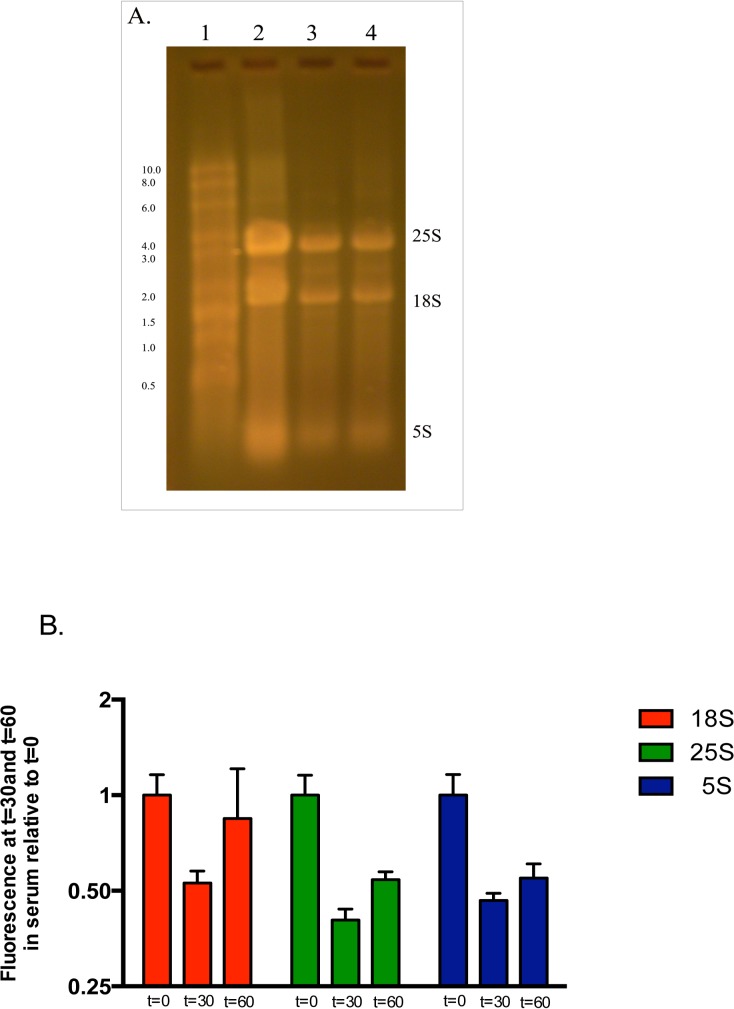
Assessments specific for ribosomal RNAs before and after hyphal stimulation in 10% FBS in H_2_O up to 60 minutes. A. RNA was gel separated and stained with SYBR Gold stain. Lane 1 has RNA size markers. 5μl of RNA were loaded in each lane as follows: Lane 2- prior to stimulation, Lane 3 after 30 min of stimulation, Lane 4 after 60 min stimulation. All three subunits are decreased by 30 min and remain so at 60 min. B. Quantitative PCR was performed on total RNA extracted before and after serum stimulation (see text). Bars represent ratios of amplicon numbers generated after stimulation to those generated prior to stimulation. Error bars represent SD. T = 0 time points were normalized to 1. Length of stimulation indicated under bars. Color scheme indicates primers specific for each ribosomal RNA sub-unit. All three subunits are decreased by 30 min and remain below baseline at 60 min.

While new classes of RNAs have been recently recognized, ribosomal RNA remains the predominant form of this molecule in eukaryotes; therefore any decrease in total RNA will likely include rRNA. Using Northern blot analysis for rRNA quantitation presents a special challenge, since the large and small subunits are usually used as controls for loading. However rRNA analysis does have one advantage that subunits can clearly be seen on stained gels and quantitative estimates can be obtained without the loss that can occur on blotting. To minimize any artifacts related to loading, assays were run in same volumes, total RNA always suspended in final volume of 50μL and the same volumes of T0 and assay pairs were carefully loaded unto gels. We are confident with our results as more than a dozen stained gels and Northern Blots gave similar results. One representative blot showing results after 30 minutes of serum exposure is shown in Fig 3A. As can be seen, both the large and the small subunits of rRNA, products of RNA polymerase I (Pol 1) are significantly diminished by 30 minutes (lanes 1, 2). 18S appears more effected on this Northern blot but this was not consistently seen and likely a blotting artifact. We did not see it on the stained gels, where both the large and small subunits can easily be seen and where additional variability of blotting is not present. Another component of the ribosome 5S RNA, a product of RNA polymerase III (Pol 3) is also diminished. Fig 3B represents the gel used in this Northern prior to blotting and stained with SYBR Gold a fluorescent dye 25–100 times more sensitive than ethidium bromide [14]. Lanes 2 and 3 represent 5μL volumes of T0 and T 30 min respectively while lanes 4 and 5 represent 1μL volumes of the same. As 25S and 18S rRNAs are easily visible one can see a decrease in RNA from T0 to T30 minutes. Densometric analysis showed similar ratios from T0 to T30 [13] for both 5 μL and 1μL volumes assuring us that our loadings are accurate. Fig 4A represents a gel loaded with RNA from T0 to T 60 minutes. As can be seen both the large and small subunits remain decreased at 60 minutes. We have also used quantitative RT-PCR (qRT-PCR) to confirm a decrease in rRNA. Fig 4B shows the combined data from qRT-PCR assays. Similar to our Northern blots all three ribosomal RNA components are decreased by 30 minutes and remain decreased at 60 minutes. This also includes 25S, while it is increasing, it is not back to baseline at 60 minutes.

### Is there a possible role for RNA in hyphal transformation?

**Fig 5:**
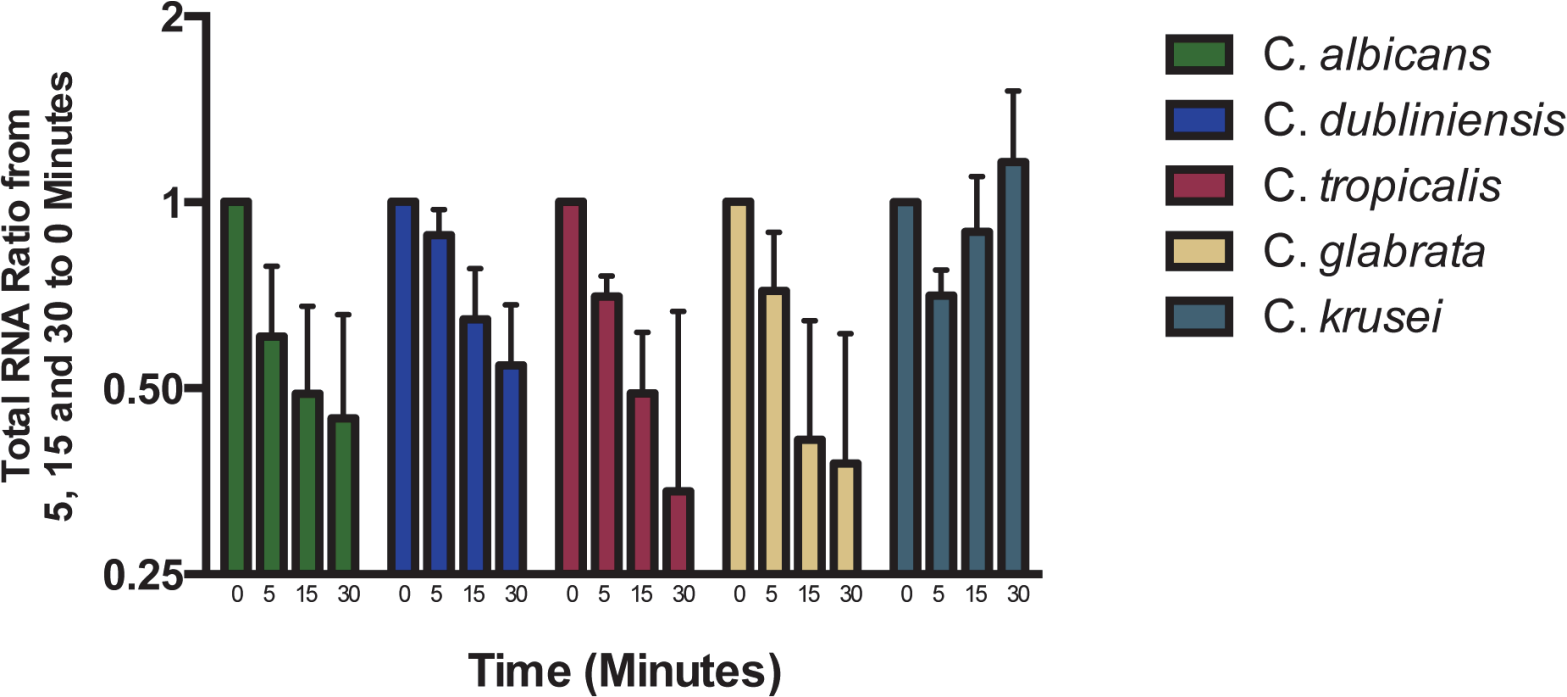
Time course of RNA content changes on hyphal stimulation in *C*. *albicans* and several other *Candida* species. Bars represent ratios of total RNA content at end of hyphal stimulation for noted time periods to RNA content prior to stimulation. Assays were done with water plus 10% FBS at 37°C. Each bar represents combined data from 2 assays and error bars represent SD.

We took several approaches to see if we could get any indication whether RNA levels have any role in hyphal transition. For RNA decrease to play such a role it would have to precede the appearance of hypha. Therefore we looked at total RNA levels as early as five minutes after start of serum exposure. As can be seen in Fig 5, total RNA in *C*. *albicans* is already significantly decreased as early as five minutes. To get to that level, one needs to assume that whatever mechanism underlies this decrease, it was activated almost immediately after 10% serum exposure.

Another approach was to compare *Candida* species with variable capacity to form hypha in 10% serum. As can be seen *C*. *tropicalis* (Fig 1G) and *C*. *dubliniensis* (Fig 1F) form germ tubes but *C*. *krusei* (Fig 1I) and *C*. *glabrata* (Fig 1H) do not. While *C*. *krusei* does not form hypha in 10% serum, it does form them on agar surface after a few days, whereas *C*. *glabrata* does not form them even on agar surface. Their data which includes both RNA content and early timing is also shown in Fig 5. They all show similar kinetics and degree of decrease of RNA as *C*. *albicans* except *C*. *krusei* which shows an initial decrease with full recovery by 30 minutes.

**Fig 6:**
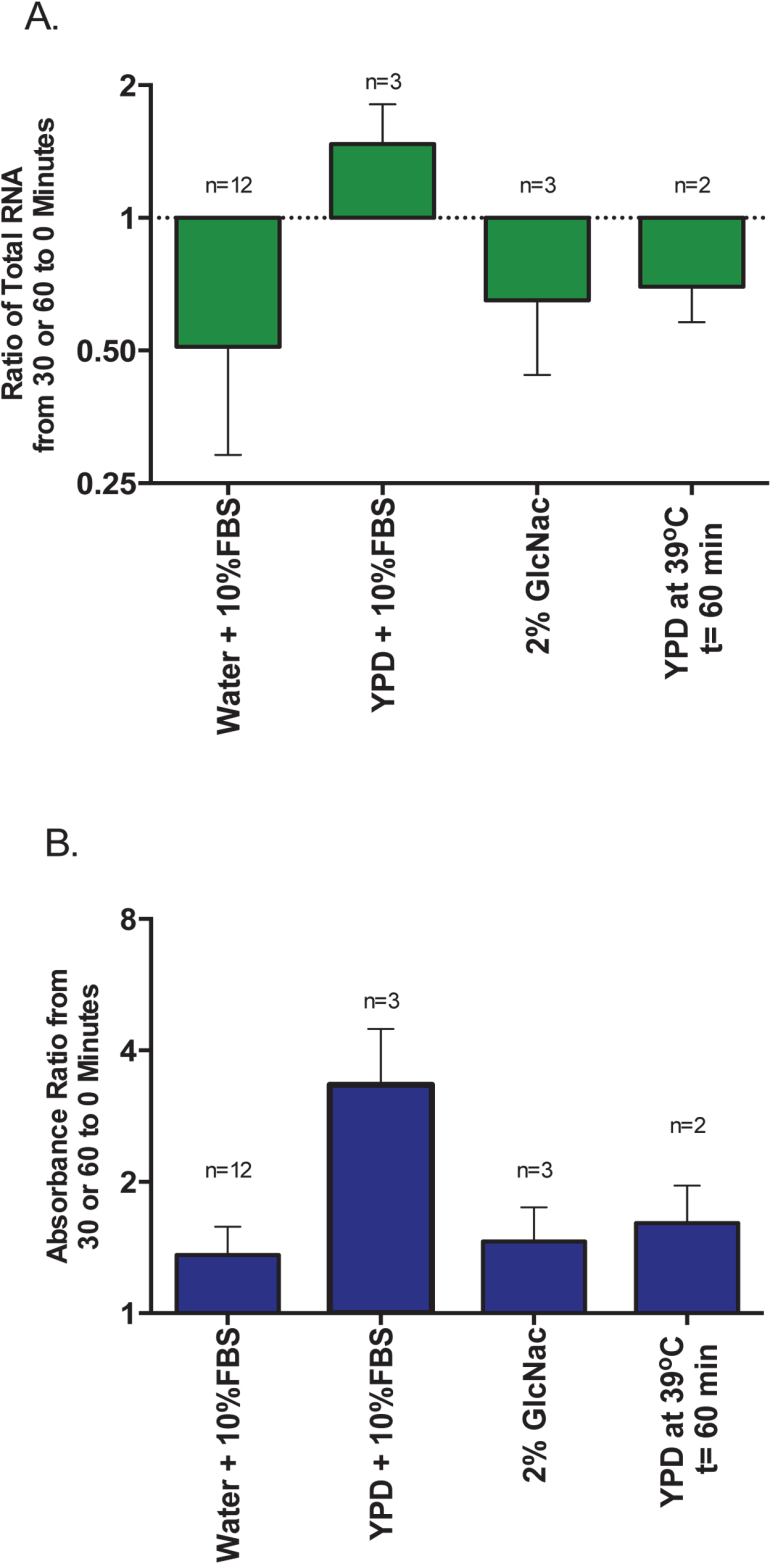
RNA content changes in *C*. *albicans* with various modes of hyphal stimulation. A. Bars represent ratios of total RNA content at end of hyphal stimulation to RNA content prior to stimulation. First bar shows the same data seen in Fig 2A (first bar) and was put in for comparison with other stimuli. Error bars represent SD. Length of assays were 30 minutes at 37°C except where otherwise noted. GlcNac stimulation reduced RNA significantly (p = 0.08) as did two degree increase in temperature but less significantly (p = 0.1). Serum in YPD enhanced total RNA content (p = 0.03). B. XTT assays run simultaneously with conditions mirroring those of part A. Bars represent ratios of absorbance after stimulation to those before stimulation. Error bars represent SD. They all show increase indicating no loss in viability or metabolic activity.

We also looked at GlcNac, a well-studied hyphal inducer that acts via Ngt1 directly activating the transcription factor Efg1, unlike serum that utilizes the MAPK and cAMP pathways [3]. The results are shown in Fig 6. Cells germinated well by 30 minutes [13] and similar to 10% serum/H_2_O there was a decrease in RNA content (Fig 6A) without loss of viability (Fig 6B).

We also did assays where nutrition for the cells was maintained. When 10% serum was added to YPD at 37°C, hyphal formation could be seen by 30 minutes. As can be seen in Fig 6A and 6B, both total RNA and formazan increased with this combination. Remarkably, when only a 2 degree increase in temperature from 37°C to 39°C in YPD was employed without any added serum, hypha were induced [13] formazan increased yet total RNA decreased (Fig 6A and 6B).

## Discussion and Conclusion

These data confirm our previous observation that rRNA is decreased on serum exposure. Additionally, GlcNac another potent hypha inducer also mirrors 10% serum both in RNA reduction and formazan formation. The obvious question this raises is whether this change in RNA plays any role in hyphal transformation? That two germination inducers also decrease rRNA and the rapidity of rRNA decrease (Fig 6), occurring prior to phenotypically observable hyphal development suggests a possibility for such a role. Also, when temperature was changed from 37°C to 39°C in YPD without any change to nutrition, hypha formed [13], formazan increased (Fig 6B) but total RNA decreased (Fig 6A), again suggesting such a role. Our assays however designed to address this point specifically, indicate that germination can proceed independent of rRNA levels. With 10% serum in H_2_O, after initial decline of RNA by 30 minutes there is no additional decline at 60 minutes (Fig 2B), yet robust germination can be observed microscopically at that point (Fig 1D). Similar results are seen with rRNA specifically (Fig 4A). When 10% serum is added to YPD, cells germinate by 30 minutes (Fig 1B), both RNA and formazan are increased (Fig 6A and 6B), again indicating that serum is capable of stimulating hyphal transformation, without a decrease in rRNA. Finally, both hypha forming and non-hypha forming *Candida* species show similar decline in RNA on 10% serum exposure, indicating this to be an independent event. Thus overall it appears that this decrease in rRNA does not play a direct causal role in hyphal formation. The possibility exists that rRNA decline potentiates hyphal transformation in a multi-step process in those that do form hypha, but since we did not do any quantitation of newly formed hypha among the various conditions, we cannot draw such a conclusion from our data.

Our data however clearly indicate that 10% serum in H_2_O seems to affect rRNA content specifically. Indeed with water alone, there was a significant decrease of total RNA and metabolic activity was also somewhat decreased by 30 minutes (Fig 2A and 2C). With 10% serum in water, at the same 30 minute period, metabolic activity was somewhat increased, hypha could easily be seen (Fig 1A), yet total RNA content is more decreased as compared to water alone (Fig 2A). At 60 minutes (Fig 2A and 2C) metabolic activity more than doubled (Fig 2A and 2C), abundant hypha were seen microscopically (Fig 1D). We also know from previous 4′, 6-diamidino-2-phenylindole (DAPI) studies [15] that hypha contain nucleic acid within septal compartments, and we have observed the same on serum induced hyphae. These details indicate that 10% serum is adequate to support nucleic acid synthesis, structural growth and metabolic activity. Yet total RNA remains decreased almost to the same level as at 30 minutes When 100% serum was used, hypha can be seen by 30 minutes (Fig 1C), metabolic activity was as good as YPD (Fig 2C), yet total RNA was decreased (Fig 2A) though to a less significant extent as compared to 10% serum.

Our data clearly shows that 10% serum in H_2_O significantly and rapidly decreases rRNA in *C*. *albicans*. Microarray studies by Nantel et al [16] profiling yeast-to-hyphal transition have not shown such a difference, but those studies were oriented toward ORF associated transcription and therefore would not detect rRNA changes. Why and by what mechanism this occurs is unknown. It is known that ribosome biogenesis is regulated in response to external signals via the mTOR kinase system, and the presence of this system in *C*. *albicans* is well established [17]. Tor 1 signaling has also been shown to play a role in hyphal development [18]. The rapid decrease in RNA suggests an active degradation of rRNA with its longer half-life. Multiple pathways for rRNA degradation in *Saccharomyces cerevisae* have been described [19]. One of them is polyadenylation of defective rRNA by TRAMP complexes setting them up for degradation by nuclear exosomes [20]. It is very likely that similar systems are also present in *C*. *albicans*. It is interesting that when we previously reported polyadenylation of both the large and small subunits of rRNA in *C*. *albicans*, we found that this polyadenylation occurred rapidly, as early as five minutes after 10% serum exposure and it came down to baseline by fifteen minutes [8,9]. This correlates well with our current findings. As seen in Fig 5, 10% serum reduces RNA rapidly, getting close to its nadir by fifteen minutes. This raises the possibility that some factor in serum predisposes even properly transcribed rRNA to be polyadenylated by the TRAMP complex causing its degradation. Finally, because of the many tandem repeats of the rRNA gene, transcription of rRNA by Poll I is very efficient, capable of producing as many as 2000 copies per minute per cell [21]. It is not unreasonable to postulate, that for more than a 50% decrease in fifteen minutes, transcription is also affected, either by some factor in serum or via mTOR, known to regulate rRNA transcription.
